# The gut microbiome and its potential role in paradoxical anaerobism in pupfishes of the Mojave Desert

**DOI:** 10.1186/s42523-020-00037-5

**Published:** 2020-05-19

**Authors:** Shrikant S. Bhute, Brisa Escobedo, Mina Haider, Yididya Mekonen, Dafhney Ferrer, Stanley D. Hillyard, Ariel D. Friel, Frank van Breukelen, Brian P. Hedlund

**Affiliations:** 1 School of Life Sciences, University of Nevada, Las Vegas, 4505 Maryland Parkway, Las Vegas, NV 89154 USA; 2 School of Dental Medicine, University of Nevada, Las Vegas, 4505 Maryland Parkway, Las Vegas, NV 89154 USA

**Keywords:** Pupfish, Ethanol metabolism, Paradoxical anaerobism, Gut microbiome, *Cetobacterium*

## Abstract

**Background:**

Pupfishes frequently enter paradoxical anaerobism in response to endogenously produced or exogenously supplied ethanol in a dose-dependent manner. To decipher the role of the gut microbiota in ethanol-associated paradoxical anaerobism, gut microbial communities were depleted using a cocktail of antibiotics and profiled using 16S rRNA gene sequencing.

**Results:**

Compared to the control group (*n* = 12), microbiota-depleted fish (*n* = 12) spent more time in paradoxical anaerobism. Our analysis indicated that the bacterial phyla *Proteobacteria*, *Fusobacteria*, *Bacteroidetes*, *Firmicutes*, *Actinobacteria*, *Patescibacteria*, and *Dependentiae* dominated the pupfish gut, which is consistent with other fish gut microbiota. Although the gut microbial communities with and without antibiotic treatment were similarly diverse, they were distinct and the greatest contribution to the dissimilarity (27.38%) was the common fish commensal *Cetobacterium*.

**Conclusions:**

This study reports the first characterization of gut microbial communities of pupfish and suggests the microbiome may play a critical role in regulating metabolic strategies that are critical for survival in extremes of temperature and oxygen concentration. We speculate that *Cetobacterium*, a primary fermenter, also consumes ethanol through secondary fermentation via an alcohol dehydrogenase and therefore regulates the transition from paradoxical anaerobism to aerobic respiration in fish. Given the wide distribution and abundance of *Cetobacterium* in warm-water fishes, this process may be of broad importance, and suggests that the microbiome be carefully considered for both conservation and aquaculture.

## Background

The Mojave Desert in the Southwestern United States is home to isolated systems of springs that are geothermally or ambiently heated to 28 to 33 °C and are inhabited by pupfishes in the genus *Cyprinodon* [1]. Previously, we demonstrated that two lineages of pupfishes reared at 33 °C frequently entered a state of paradoxical anaerobism [2, 3]. For periods of as much as 149 min, these pupfish consumed negligible oxygen despite its availability. Instead, pupfish reared or acclimated to 33 °C produced ~ 7.3X more ethanol than their counterparts maintained at 28 °C. Pupfish spontaneously exited paradoxical anaerobism, indicating there are no lasting deleterious effects on mitochondrial function. Both 28- and 33 °C-acclimated pupfish entered paradoxical anaerobism when exposed to exogenous ethanol in a dose-dependent manner [2]. Although 28 °C-acclimated pupfish rarely spontaneously used paradoxical anaerobism (1/262 assayed fish measured for a 2 h period), exposure to 1% ethanol (vol/vol) resulted in seven out of 12 of the fish immediately entering paradoxical anaerobism. We proposed that ethanol metabolism and the resulting accumulation of acetaldehyde results in closure of voltage-dependent anion channels (VDACs) in living pupfish and ultimately, the phenotype of paradoxical anaerobism [2] (see discussion for details). If our model is correct, changes in ethanol metabolism should affect use of paradoxical anaerobism. Our earlier work measuring ethanol accumulation by 28 °C- and 33 °C-acclimated fish suggested a microbial interaction. Measuring ethanol accumulation in a sealed container with fish required us to pre-boil aquarium water, rinse the fish, and ensure the fish did not defecate or swim vigorously during the assay; rapid reduction of exogenous ethanol in the water otherwise occurred [2].

In the present study, we explore the possibility that the pupfish gut microbiome plays a role in the regulation of paradoxical anaerobism. Specifically, if the microbiome served as a sink for endogenously produced ethanol, then reducing the autochthonous gut microbiome with antibiotic treatment would be predicted to enhance the expression of paradoxical anaerobism produced by exogenous exposure to ethanol. In this study, gut contents of fish treated with antibiotics and untreated controls were collected for analysis of microbial community diversity and composition, which were then related to the expression of paradoxical anaerobism.

## Results

### Exposure to antibiotics led to increased time in paradoxical anaerobism

**Fig. 1:**
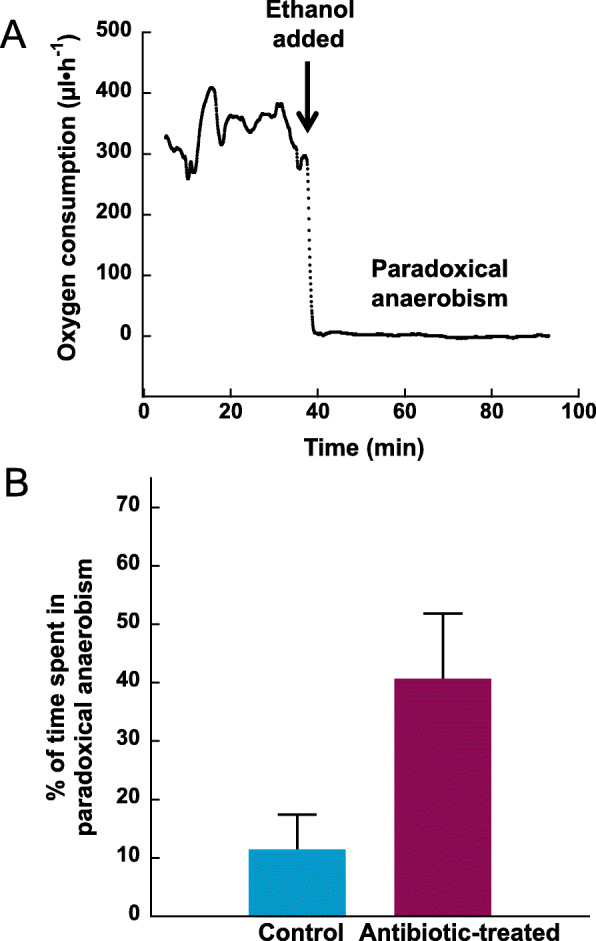
Measurements of paradoxical anaerobism. **a** Demonstration of induction of paradoxical anaerobism using 1% ethanol (vol/vol). Pupfish (*n* = 12 antibiotic-treated pupfish and 12 control pupfish) were placed in the metabolic chamber and oxygen consumption was monitored. At the time indicated by the arrow, ethanol was added to a final concentration of 1% (vol/vol). In this example, an antibiotic-treated pupfish immediately entered paradoxical anaerobism and remained in that metabolic state for the duration of the experiment. **b** Following addition of ethanol, pupfish oxygen consumption was monitored for ~ 1 h. Data are means ± SE of the % of that time which was spent with negligible oxygen consumption. Values are significantly different (Welch’s t-test, *p* = 0.037)

Exposure to a cocktail of antibiotics (kanamycin, gentamicin, colistin, metronidazole, and vancomycin) made fish more prone to entering paradoxical anaerobism (Fig.1). In Fig.1a, an example of paradoxical anaerobism use is provided. Immediately after introduction of ~ 1% ethanol (vol/vol) to the aquarium water, the fish entered a prolonged period of negligible oxygen use. Pupfish exposed to antibiotics and ethanol spent 40.5 ± 11.3% of the post-ethanol time in paradoxical anaerobism - approximately 3.6X longer than did control fish (Fig.1b). This difference was statistically significant (Welch’s t-test, *p*-value = 0.037). All 12 antibiotic-treated, 28 °C-acclimated pupfish used some paradoxical anaerobism when exposed to 1% ethanol (vol/vol), whereas 8 of the 12-control pupfish used paradoxical anaerobism, which is consistent with our previous results [2].

### Antibiotic treatment was associated with the changes in community composition

**Fig. 2:**
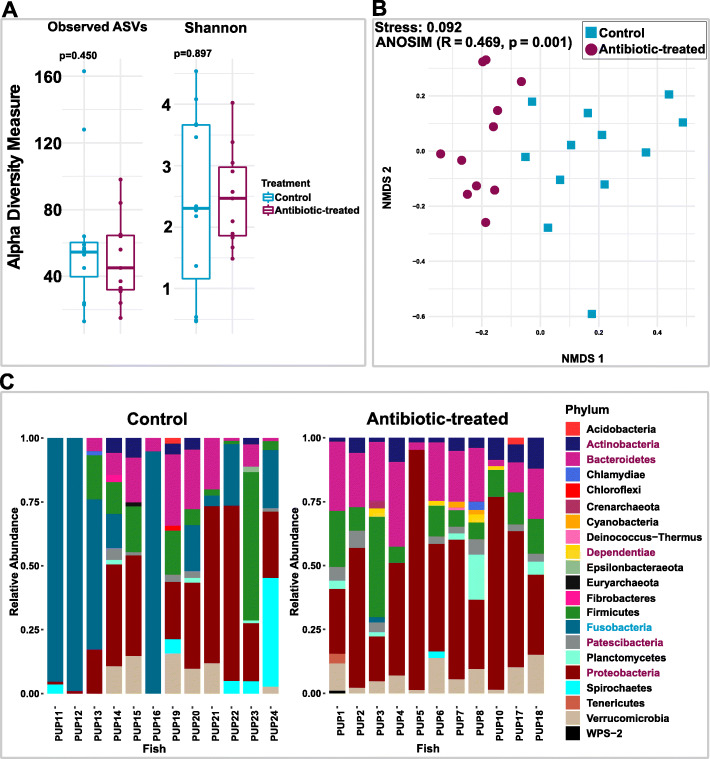
Microbial diversity analysis. **a** Box-and-whisker plots of alpha diversity metrics (Observed ASVs and Shannon index). Each box represents interquartile range; the line inside the box is a median. **b** Bray–Curtis dissimilarity-based non-metric multidimensional scaling (NMDS) plot showing the distribution of antibiotic-treated and control fish samples in ordination space. **c**) Bar plot showing the relative abundance (> 1%) of gut microbiota at the phylum level. Five phyla indicated in cherry-red color in legend were significantly higher (Welch’s t-test, *p* < 0.05) in antibiotic-treated fish, whereas the one indicated in cyan (*Fusobacteria*) was significantly depleted (Welch’s t-test, *p* < 0.05). The phylum *Fusobacteria* was dominated by genus *Cetobacterium*

Illumina sequencing yielded an average of 18,688 (range: 3713 – 82,969) 16S rRNA gene sequences per fish gut sample (Supplementary Table 1). One of the samples, PUP9, from the antibiotic-treated group, was removed from further analysis due to low sequence count. The microbiomes were moderately diverse, with amplicon sequence variant (ASV) richness ranging from 11 to 154 and Shannon diversity values ranging from 0.67–6.23, and there was no significant difference in richness or diversity (Observed ASVs and Shannon index, Fig.2a; Table S1) between control and antibiotic-treated microbiomes (Welch’s t-test, observed ASVs: *p* = 0.450 and Shannon index: *p* = 0.897). However, beta diversity analysis based on non-metric multidimensional scaling (NMDS) using Bray-Curtis dissimilarity indicated that antibiotic treatment changed the composition of the microbiome (Fig.2b). This was further demonstrated by ANOSIM (Analysis of Similarity) analysis (*R* = 0.469, *p* = 0.001). Overall, the dominant phyla were *Proteobacteria* (29.9 and 46.9%), *Fusobacteria* (47.3 and 2.0%), *Bacteroidetes* (11.7 and 18.5%), *Firmicutes* (15 and 13.8%), *Actinobacteria* (4.8 and 5.3%), *Patescibacteria* (2.5 and 4.1%), and *Dependentiae* (0.0 and 2.3%); numbers in parentheses indicate mean relative abundance of each phylum in control and antibiotic-treated groups, respectively. Out of the 23 phyla detected in all samples (Fig.2c), *Proteobacteria, Bacteroidetes, Actinobacteria*, *Patescibacteria*, and *Dependentiae* had higher relative abundance in the antibiotic-treated group (Welch’s t-test, *p* < 0.05), whereas only *Fusobacteria* was significantly reduced due to antibiotic treatment (Welch’s t-test, *p* < 0.05).

**Fig. 3:**
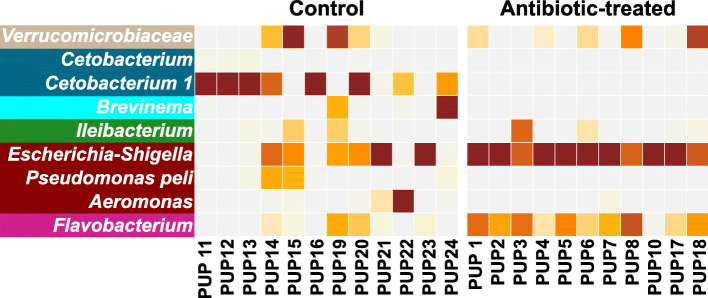
Heatmap displaying top ASVs responsible for dissimilarity between control and antibiotic-treated fish. Heatmap of the relative abundance of 09 ASVs (genus- or family-level assignments are indicated) identified using SIMPER analysis. These taxa contributed cumulatively to 50% of community dissimilarities. Each square represents the relative abundance of the given ASV, higher intensity of the brown color correlates with high relative abundance. Taxa are color-coded as per the phylum-level assignments as: *Verrucomicrobia*: orange; *Fusobacteria*: yellow; *Spirochetes*: pink; *Firmicutes*: sky-blue; *Proteobacteria*: green, and *Bacteroidetes*: grey

SIMPER (SIMilarity of PERcentages) analysis was performed to determine the contribution made by specific ASVs to the observed dissimilarity between control and antibiotic-treated fish. SIMPER identified nine ASVs contributing to 50% of observed differences, of which *Cetobacterium* (*Fusobacteria*), *Brevinema* (*Spirochaetes*), *Pseudomonas pseudomonaspeli* (*Proteobacteria*), *Ileibacterium* (*Firmicutes*), *Aeromonas* (*Proteobacteria*), and *Verrucomicrobiaceae* (*Verrucomicrobia*) were enriched in the control group, while *Escherichia-Shigella* (*Proteobacteria*) and *Flavobacterium* (*Bacteroidetes*) ASVs were enriched in the antibiotic-treated group (Fig.3; Supplementary Table 2). ANCOM (Analysis of Composition of Microbiomes) (W = 719) suggested that the loss of *Cetobacterium* in antibiotic-treated fish as a strong indicator of community differences. *Cetobacterium* was represented by two different ASVs, designated *Cetobacterium* and *Cetobacterium* 1 (Fig.3), which represent either separate species or separate populations of a single species. However, the genetic distance between these ASVs cannot be accurately assessed by small 16S rRNA gene fragments, alone. The *Cetobacterium* 1 ASV comprised 33% of the healthy pupfish gut microbiome, whereas the *Cetobacterium* ASV was present at low abundance; neither were detected in antibiotic-treated fish (Fig.3).

### *Cetobacterium* may be involved in ethanol metabolism

**Fig. 4:**
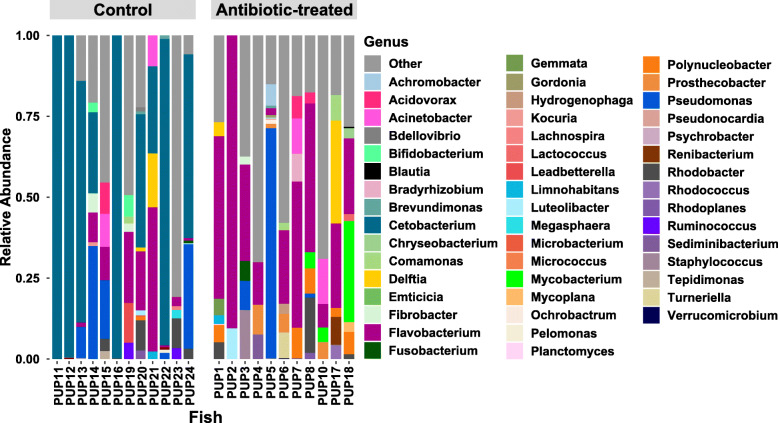
Genus-level relative abundance of 129 ASVs that are predicted to encode alcohol dehydrogenase. These ASVs are predicted to have alcohol dehydrogenase activity. ASVs with no predicted alcohol dehydrogenase are indicated as Other (red)

To probe the possible mechanism of microbial mediation of paradoxical anaerobism, we identified microbial taxa contributing to ethanol metabolism by predicting the presence of alcohol dehydrogenase in the simulated metagenome using PICRUSt (Phylogenetic Investigation of Communities by Reconstruction of Unobserved States). We identified 129 ASVs that likely have an alcohol dehydrogenase gene (*adh*; KO: K00001). Notably, ASVs assigned to *Cetobacterium* were the dominant bacteria predicted to encode Adh in control fish, indicating the potential role of *Cetobacterium* in clearing ethanol reaching the gut environment in these fish (Fig.4). In contrast, *Flavobacterium* ASVs were the dominant bacteria to predicted to encode Adh in antibiotic-treated fish. Other taxa that contributed to the *adh* gene pool include members of the *Actinobacteria* (*Bifidobacterium and Mycobacterium*)*; Proteobacteria* (*Acinetobacter, Delftia, Polynucleobacter,* and *Pseudomonas*)*;* and *Firmicutes*.

## Discussion

Voltage-dependent anion channels (VDACs) allow substrates to enter the outer mitochondrial membrane and are assumed to be open during normal metabolism [4–6]. This assumption has led many to believe that low molecular weight molecules pass freely through the outer mitochondrial membrane. The continuation of glycolysis is dependent on the availability of NAD^+^ [7]. In mammals, the production of lactate allows for NAD^+^ regeneration during anaerobic metabolism. Under hypoxia, goldfish and other warm-acclimated fishes, may also regenerate NAD^+^ through production of ethanol [2, 8]. For instance, anoxic exposure of goldfish to 12 h at 4 °C results in excretion of 6.63 μmol ethanol • g^− 1^ fish into the water and tissue accumulation of 4.58 μmol ethanol • g^− 1^ fish. However, ethanol metabolism, resulting in the production of acetaldehyde, is also able to suppress oxygen consumption through closure of the VDACs [9]. A closed VDAC would limit substrate entry into the mitochondrion and thus, oxygen consumption. We believe that paradoxical anaerobism is the result of pupfish lapsing into this phenotype e.g., relatively placid pupfish produce some ethanol as part of their normal metabolism. The associated acetaldehyde closes some VDACs. While this phenotype is frequently manifested by oxygen consumption that is more variable and lower than what may be otherwise expected, we believe a positive feedback mechanism resulting in more and more closure of the VDACs results in the paradoxical anaerobism characterized by negligible oxygen consumption. Consistent with this model are data from 33 °C-acclimated pupfish demonstrating a much higher frequency of a state of variable oxygen consumption (~ 20% of 33 °C-acclimated pupfish assayed for 2 h as compared to < 4% of 28 °C-acclimated pupfish), lower than expected oxygen consumption, and lower aerobic scope (maximum oxygen consumption • rVO_2_^− 1^) [2, 3]. In other words, both 28- and 33 °C-acclimated pupfish are likely using a mosaic of both paradoxical anaerobism and oxygen consumption. However, the use of paradoxical anaerobism is more extensive in the 33 °C-acclimated pupfish than their 28 °C-acclimated counterparts. As a result, these pupfish are more likely to lapse into extensive paradoxical anaerobism use characterized by negligible oxygen consumption.

We hypothesized that the gut microbiome would serve as a sink for ethanol since ethanol readily crosses membranes. Consistent with our model is the finding here that pupfish exposed to antibiotics and ethanol experienced 3.6X more paradoxical anaerobism than did the control fish (Fig.1b). We contend that depletion of the microbiome results in more circulating ethanol in the pupfish and therefore longer bouts of paradoxical anaerobism. To gain insight into microorganisms that might be responsible for ethanol consumption, we characterized the gut microbial communities of pupfish treated with the antibiotic cocktail and compared them to control fish. Consistent with other fish gut microbiomes, the pupfish microbiome was dominated by members of the *Proteobacteria*, *Fusobacteria*, *Bacteroidetes, Firmicutes*, and *Actinobacteria* [10]. Yet, there were significant differences in microbial communities between the control and antibiotic-treated pupfish populations, which correlated with the increase in paradoxical anaerobism in the antibiotic-treated fish.

Prophylactic use of antibiotics during fish farming has been linked with increased abundance of antibiotic-resistant members of *Bacteroidetes* and *Proteobacteria* [11, 12], which is consistent with our observation of increased abundance of *Bacteroidetes* (*Flavobacterium*) and members of *Proteobacteria*, specifically *Enterobacteriaceae*, in antibiotic-treated pupfish. Species of the genus *Flavobacterium* (*F. psychrophilum*, *F. columnare*, and *F. branchiophilum*) have been described in localized and systemic infections and implicated in mortality of both wild and aquaculture fishes [13]. Antibiotic-resistant *Flavobacterium* isolates have also been obtained from fish farms [14], broadly consistent with their increased abundance in this study.

**Fig. 5:**
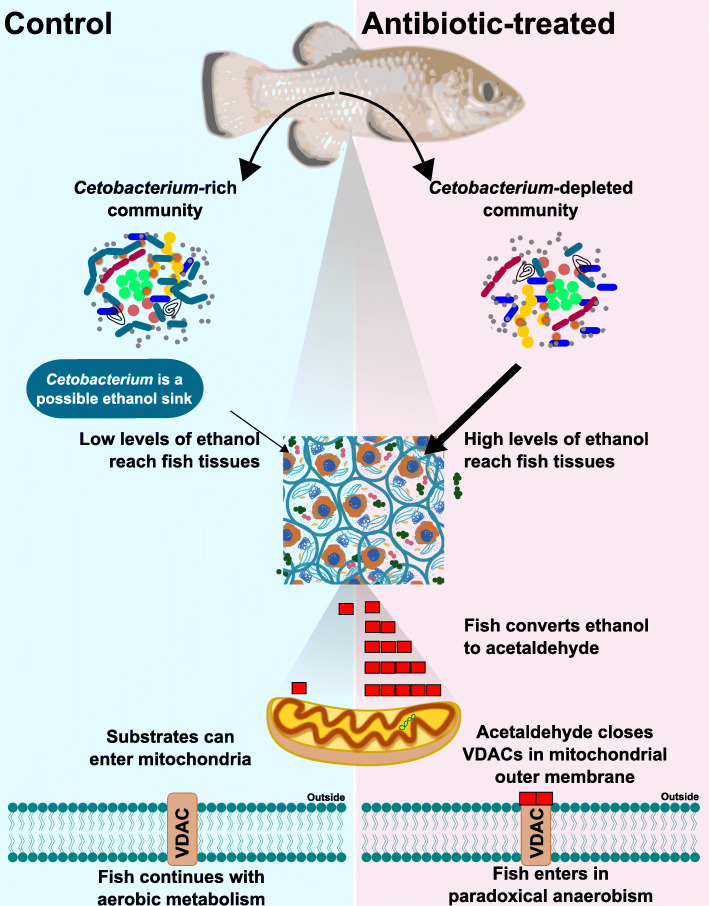
Summary of the results and hypothesis. We propose that the *Cetobacterium* in control pupfish may be acting as an ethanol sink. Thus, depleting *Cetobacterium* using the antibiotic cocktail might cause increased accumulation of the acetaldehyde in fish tissues leading to closure of VDAC and eventually paradoxical anaerobism

Elimination of *Fusobacteria*, more specifically *Cetobacterium*, from the antibiotic-treated fish indicates general sensitivity of this microorganism to the antibiotic cocktail used in this study. *Cetobacterium ceti* and other species of the phylum *Fusobacteria* are broadly sensitive to antibiotics, with the notable exception of vancomycin [15]. *Cetobacterium* is a microaerotolerant primary fermenter of carbohydrates and peptides, producing acetate as the major fermentation product, and is particularly abundant in warm-water fishes such as tilapia [16], carp [17], arapaima [18], catfish, and bass [19], comprising > 70% of the gut microbial community in many individuals. Isolates of *Cetobacterium* produce large quantities of vitamin B12 and is speculated to therefore promote fish health [20]. However, we suggest an additional hypothesis that *Cetobacterium* may play a role in regulating ethanol toxicity and the balance between paradoxical anaerobism and aerobic respiration in fish that are often limited for dissolved oxygen (Fig.5). Several pathways for microbial ethanol consumption are known [21, 22], but all likely involve use of some form of Adh. Our finding that *Cetobacterium* is the dominant organism predicted to encode Adh in healthy pupfish and its absence in antibiotic-treated fish with prolonged bouts of paradoxical anaerobism is consistent with this alternative hypothesis; however, a rigorous test of this would require a direct test, for example by applying the principles of Koch’s postulates.

Of direct relevance to active conservation efforts with pupfishes, our surveys of planktonic cells in springs across Ash Meadows National Wildlife Refuge show that low-abundance populations of *Cetobacterium* are ubiquitous, which is consistent with a separate report of low-abundance planktonic *Cetobacterium* in Devils Hole [23]. In contrast, *Cetobacterium* is absent from nearby springs without fish populations. These planktonic bacteria are likely indicators that *Cetobacterium* is present in the guts of inhabitant pupfish and serve as inoculum for developing fish. Other work has suggested that *Cetobacterium* may be a target for probiotic development for fish well-being [19, 24]. Our work suggests that *Cetobacterium* might be important for the development and health of pupfish and should be considered within the context of the current practice of antibiotic treatment of pupfish eggs at the AMFCF [25].

## Conclusion

Our work catalogues the gut microbial communities of pupfishes of the Mojave Desert and proposes its involvement in the paradoxical anaerobism observed in these fish. We show that removal of dominant gut flora, including members of genus *Cetobacterium* using antibiotics leads to increased duration of paradoxical anaerobism which could be linked with the ethanol utilizing abilities of *Cetobacterium* through the action of alcohol dehydrogenase (Fig.4). We further propose that regular microbiome monitoring of Ash Meadow Fish Conservation Facility (AMFCF) to detect the presence of *Cetobacterium* in the refuge tank, and the fish living in it, may help the active conservation of pupfish.

## Methods

### Fish husbandry and antibiotic treatment

Refuge pupfish that have been described as *Cyprinado diabolis* and *Cyprinadon nevadensis mionectes* hybrids [26] were maintained at 28 °C as described previously [27]. Pupfish were either left untreated (*n* = 12) or exposed (*n* = 12) to a cocktail of kanamycin (400 μg • ml^− 1^), gentamicin (35 μg • ml^− 1^), colistin (850 IU • ml^− 1^), metronidazole (215 μg • ml^− 1^), and vancomycin (45 μg • ml^− 1^) for 48 h prior to oxygen consumption measurement. This combination of broad-spectrum antibiotics acting on protein synthesis (kanamycin and gentamicin), cell membrane integrity (colistin), DNA synthesis (metronidazole) and peptidoglycan synthesis (vancomycin) is often used to deplete the mouse gut microbial community to study *Clostridioides difficile* infections and is highly effective [28].

### Measurement of oxygen consumption after ethanol treatment

Routine rates of oxygen consumption (rVO_2_) were measured in fish showing minimal activity using flow-through respirometry (Fig.S1) [2, 3]. Briefly, four fish at a time were placed individually in glass metabolic chambers (60 mL volume) that were connected to flow cells with Clark-type oxygen electrodes (Strathkelvin, Scotland). The apparatus was placed in a ~ 40 L aerated and heated (28 °C) aquarium. Water was drawn into the metabolic chambers from the surrounding aquarium and through the flow cells using a peristaltic pump. Oxygen consumption was calculated with Strathkelvin software for flow-through respirometry using the appropriate temperature and pressure corrections. Flow rates were adjusted, according to the size of the fish, to maintain the excurrent PO_2_ above 100 mmHg (13.32 kPa; and well above the critical PO_2_ value of 47 mmHg that would otherwise limit oxygen consumption) [2]. These flow rates were ~ 6 to 8.4 mL • min^− 1^.

Each trial began with a period of ~ 60 min after fish were placed in the chambers to allow rVO_2_ to stabilize. Four hundred mL of 95% ethanol (prepared from 100% anhydrous ethanol) was added to the 40 L aquarium to give a final concentration of ~ 1% ethanol (vol/vol). After ~ 60 min, each fish was removed, and the electrode was allowed to return to ambient PO_2_ to allow for any correction of electrode drift. As a result, the exact time for each trial varied from 56 to 63 min and data are expressed as a percentage of that time to allow for ready comparison. At the end of each trial, fish were anesthetized in MS 222 and their intestines removed, sliced longitudinally, and the contents removed by gentle scraping. The removed contents were frozen at − 80 °C until analysis.

### Microbial community profiling

The intestinal contents of each fish were homogenized at room temperature in a microcentrifuge tube using a sterile pestle (Fisher Scientific, cat # 12–141-364) and used for DNA extraction. The metagenomic DNA was extracted from intestinal contents of each fish using the QIAamp Fast DNA Stool Mini Kit and quantified using NanoDrop ND-1000. Microbial communities were profiled using V4 region of 16S rRNA gene and the sequencing was performed at Argonne National Laboratory. Briefly, V4 region of the 16S rRNA gene was PCR-amplified using modified primers 515F: GTGYCAGCMGCCGCGGTAA and 806R: GGACTACNVGGGTWTCTAAT, with the forward primer containing a 12 bp barcode [29]. The PCR products were cleaned, quantified and pooled at equimolar concentration. Paired-end sequencing of pooled amplicons was performed using MiSeq platform and customized sequencing primers.

### Data analysis

A Welch’s t-test administered in R was used to compare mean paradoxical anaerobism time in control and antibiotic-treated groups.

All sequence-based analysis was performed in QIIME2 (version 2018.6) [30] and the resulting data were exported for further analysis and visualization in R [31]. Raw Illumina reads were demultiplexed using sample-specific barcodes, denoised using DADA2 [32], and clustered into 938 high-quality ASVs. The DADA2 denoising approach was used to cluster sequences into ASVs as it can differentiate between true biological sequences from sequences containing errors introduced during amplification and sequencing, and simultaneously provides higher taxonomic resolution [32]. Taxonomy was assigned to each ASV using a naïve Bayesian classifier trained on the V4 region of 16S rRNA gene and the ‘Silva 132 99% OTUs full-length sequences’ database. ANOSIM analysis was performed to identify differences in antibiotic-treated and control group microbiomes. SIMPER analysis [33] was used to identify ASVs responsible for between-group differences. ANCOM analysis [34] was used to identify differentially abundant taxa.

Additionally, to assess the contribution of microbiome in ethanol metabolism, the potential microbial metagenome profile was obtained using PICRUSt [35]. For this, DADA2-denoised sequence data were used to pick OTUs (close-reference) using the VSEARCH algorithm and identified using the Greengenes database (version 13.5). The resulting OTU table was used as an input for PICRUSt analysis at http://galaxy.morganlangille.com/.

## Supplementary information

**Additional file 1: Figure S1.** Measurement of oxygen consumption. A schematic showing the arrangement of a glass metabolic chamber containing fish, an oxygen electrode, and peristaltic pump used to measure the oxygen consumption by pupfish.

**Additional file 2: Table S1.** Sample Metadata. **Table S2.** SIMilarity PERcentage (SIMPER) analysis based on Bray-Curtis dissimilarity between antibiotic-treated and control fish. Given is the mean relative abundance in each group and cumulative contribution of nine SVs contributing towards 50% of dissimilarity between the groups.

## Data Availability

The raw sequences are deposited in the NCBI Sequence Read Archive (SRA) under the BioProject accession PRJNA561361. All scripts used to process the 16S rRNA gene sequence data have been deposited in GitHub at https://github.com/hedlundb/PupFish

## Abbreviations

VDACs: Voltage-Dependent Anion Channels
rVO2: Rates of oxygen consumption
V4 region of 16S rRNA: Variable region 4 of 16S ribosomal ribonucleic acid
bp: Base pairs
PCR: Polymerase chain reaction
QIIME2: Quantitative insights into microbial ecology 2
DADA2: Divisive amplicon denoising algorithm 2
ASVs: Amplicon sequence variants
OTUs: Operational taxonomic units
NMDS: Nonlinear multidimensional scaling
ANOSIM: Analysis of similarity
SIMPER: Similarity Percent
ANCOM: Analysis of composition of microbiomes
VSEARCH: Vectorized search
PICRUSt: Phylogenetic investigation of communities by reconstruction of unobserved States
Adh: Alcohol dehydrogenase
*adh*: Alcohol dehydrogenase gene
NCBI: National center for biotechnology information
SRA: Sequence read archive
NAD^+^: Nicotinamide adenine dinucleotide
