# Supplementary material for: The gut microbiome and its potential role in paradoxical anaerobism in pupfishes of the Mojave Desert
Source: Anim Microbiome. 2020 May 19;2:20. doi: 10.1186/s42523-020-00037-5 (PMC7807710; doi:10.1186/s42523-020-00037-5)
Supplement: Supplementary file 1 — Additional file 1: Figure S1. Measurement of oxygen consumption. A schematic showing the arrangement of a glass metabolic chamber containing fish, an oxygen electrode, and peristaltic pump used to measure the oxygen consumption by pupfish. [file 42523_2020_37_MOESM1_ESM.pdf]

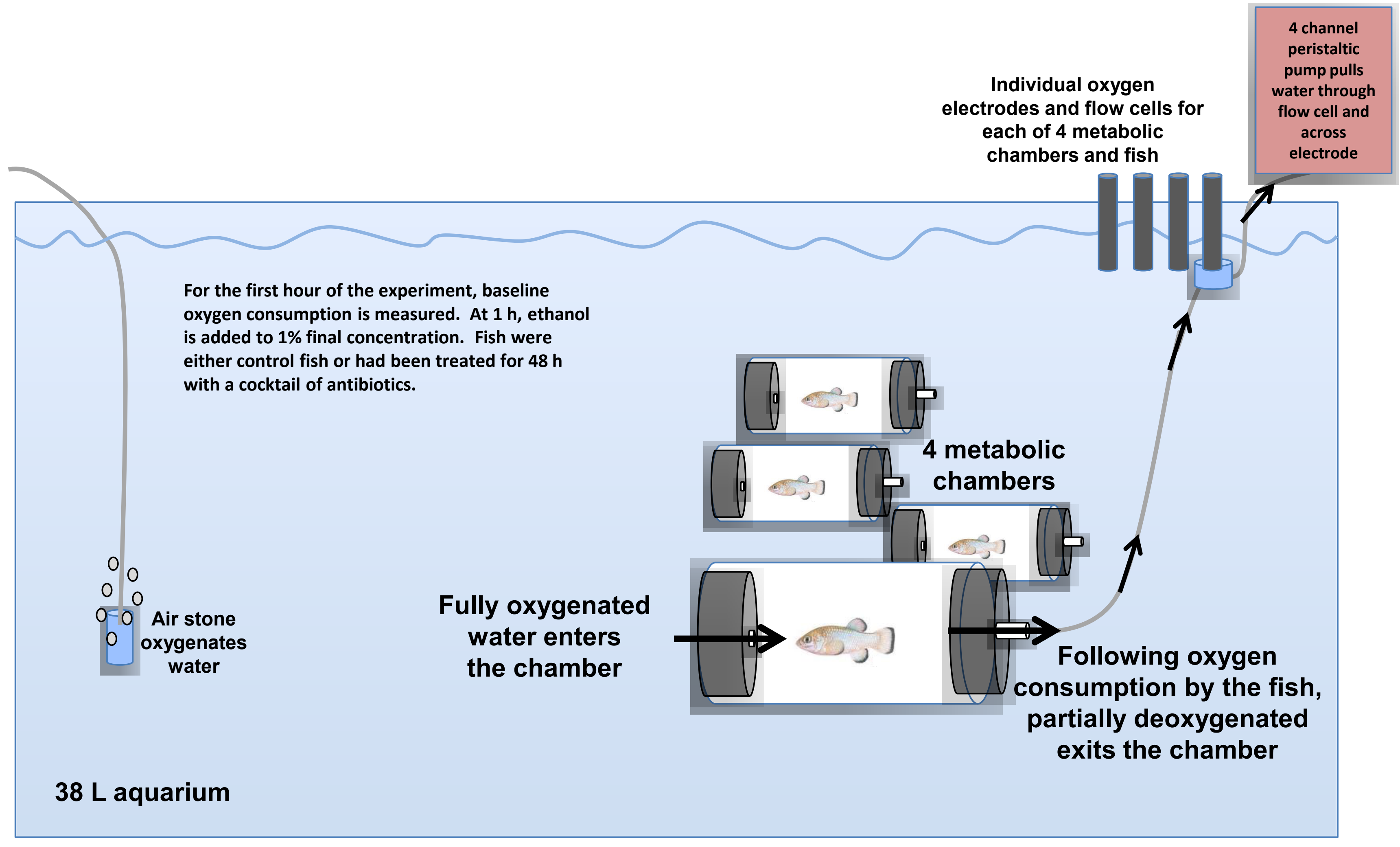
4 channel peristaltic pump pulls water through flow cell and across electrode
Individual oxygen electrodes and flow cells for each of 4 metabolic chambers and fish
For the first hour of the experiment, baseline oxygen consumption is measured. At 1 h, ethanol is added to 1% final concentration. Fish were either control fish or had been treated for 48 h with a cocktail of antibiotics.
4 metabolic chambers
Fully oxygenated water enters the chamber
Air stone oxygenates water
Following oxygen consumption by the fish, partially deoxygenated exits the chamber
38 L aquarium
